# Treatment patterns and survival outcomes of patients admitted to the intensive care unit due to immune‐related adverse events of immune checkpoint inhibitors

**DOI:** 10.1002/cam4.7302

**Published:** 2024-06-20

**Authors:** Lishi Lin, Aletta P. I. Houwink, Jolanda M. van Dieren, Esther K. Wolthuis, Johannes V. van Thienen, Michiel S. van der Heijden, John B. A. G. Haanen, Jos H. Beijnen, Alwin D. R. Huitema

**Affiliations:** ^1^ Department of Pharmacy & Pharmacology The Netherlands Cancer Institute‐Antoni van Leeuwenhoek Hospital Amsterdam The Netherlands; ^2^ Department of Anaesthesiology and Intensive Care The Netherlands Cancer Institute‐Antoni van Leeuwenhoek Hospital Amsterdam The Netherlands; ^3^ Department of Gastrointestinal Oncology The Netherlands Cancer Institute‐Antoni van Leeuwenhoek Hospital Amsterdam The Netherlands; ^4^ Department of Medical Oncology The Netherlands Cancer Institute‐Antoni van Leeuwenhoek Hospital Amsterdam The Netherlands; ^5^ Department of Molecular Oncology and Immunology The Netherlands Cancer Institute‐Antoni van Leeuwenhoek Hospital Amsterdam The Netherlands; ^6^ Department of Clinical Oncology Leiden University Medical Center Leiden The Netherlands; ^7^ Department of Pharmaceutical Sciences Utrecht University Utrecht The Netherlands; ^8^ Department of Pharmacology Princess Máxima Center for Pediatric Oncology Utrecht The Netherlands; ^9^ Department of Clinical Pharmacy University Medical Center Utrecht, Utrecht University Utrecht The Netherlands

**Keywords:** immune checkpoint inhibitors, immune‐related adverse events, intensive care unit, survival

## Abstract

**Introduction:**

Severe immune‐related adverse events (irAEs) due to immune checkpoint inhibitors (ICIs) can lead to admission to the intensive care unit (ICU). In this retrospective study, we determined the incidence, treatment patterns and survival outcomes of this patient population at a comprehensive cancer center.

**Methods:**

All patients admitted to the ICU due to irAEs from ICI treatment between January 2015 and July 2022 were included. Descriptive statistics were reported on patient characteristics and treatment patterns during hospital admission. Overall survival (OS) from the time of ICU discharge to death was estimated using the Kaplan–Meier method.

**Results:**

Over the study period, 5561 patients received at least one ICI administration, of which 32 patients (0.6%) were admitted to the ICU due to irAEs. Twenty patients were treated with anti‐PD‐1 plus anti‐CTLA‐4 treatment, whereas 12 patients were treated with ICI monotherapy. The type of irAEs were de novo diabetes‐related ketoacidosis (*n* = 8), immune‐related gastrointestinal toxicity (*n* = 8), myocarditis or myositis (*n* = 10), nephritis (*n* = 3), pneumonitis (*n* = 2), and myelitis (*n* = 1). The median duration of ICU admission was 3 days (interquartile range: 2–6 days). Three patients died during ICU admission. The median OS of the patients who were discharged from the ICU was 18 months (95% confidence interval, 5.0—NA).

**Conclusion:**

The incidence of irAEs leading to ICU admission in patients treated with ICI was low in this study. ICU mortality due to irAEs was low and a subset of this patient population even had long‐term survival.

## 1 INTRODUCTION

Ipilimumab was the first immune checkpoint inhibitor (ICI) receiving drug approval in 2011 for the treatment of metastatic melanoma.^1^ Since then, multiple ICIs have been approved for a variety of advanced cancer types, and this number will likely continue to increase in the future.^2^ These ICIs have revolutionized the field of cancer treatment, and ICI treatment is shifting from later‐line treatment options to earlier disease settings.^3^ In addition, ICI are gaining ground as adjuvant and neoadjuvant treatment options in early‐stage cancer.^4^, ^5^, ^6^, ^7^, ^8^

It is estimated that one third of patients with cancer is eligible for ICI treatment based on data from the US, whereas with the ongoing developments the number of patients who will receive ICI treatment in future will likely increase.^9^ As a consequence, the number of patients experiencing adverse events will also increase.^10^ A unique type of adverse event associated with the use of ICIs is the class of immune‐related adverse events (irAEs). These irAEs are autoimmune conditions that can affect any organsystem in the body.^11^

The European Society of Medical Oncology released a clinical practice guideline on the management of ICI‐induced irAEs, in which corticosteroids are recommended as first‐line treatment, followed by other immunosuppressive therapies depending on the severity of the irAEs and organ systems involved.^12^ In severe cases, hospitalization is required to treat irAEs, whereas intensive care unit (ICU) admissions can be required to monitor and support organ functions in life‐threatening situations.

Only a limited number of studies have been conducted to specifically determine the treatment course and survival outcome of patients hospitalized due to irAEs, which is especially true for patients admitted to the ICU.^13^, ^14^, ^15^, ^16^ Therefore, this retrospective study aimed to determine the incidence, treatment patterns, and survival outcomes of patients administered to the ICU of a comprehensive cancer center as a result of ICI‐induced toxicity.

## 2 METHODS

This retrospective study was performed at the Netherlands Cancer Institute—Antoni van Leeuwenhoek Hospital (NKI‐AvL), Amsterdam, the Netherlands, which is a comprehensive cancer center. The ICU of the NKI‐AvL is classified as a level 2 ICU according to the report of the task force of the World Federation of Societies of Intensive and Critical Care Medicine.^17^ Our ICU is an eight bed facility, which is the only monitoring facility in our hospital since there is no cardiac care unit or emergency department.

The total number of patients who received at least one administration of atezolizumab, avelumab, cemiplimab, dostarlimab, durvalumab, ipilimumab, nivolumab, or pembrolizumab at our institute between January 2015 and July 2022 was determined. Patients aged 18 years or older were included in this study if they were admitted to the ICU due to irAEs of ICIs. Patients who did not consent to their data being used for research were excluded. No other exclusion criteria were applied. The assessment whether ICU admittance was due to irAEs was performed by the treating physician and reviewed by investigators (LL and AHo). In case a patient experienced more than one type of irAE, distinction was made between the irAE contributing most to ICU admission and additional irAE. Gastrointestinal irAEs were confirmed by biopsies as part of the standard of care. Other types of irAEs, such as nephritis, were not confirmed by biopsies as part of the standard of care.

In case of gastrointestinal irAE and nephritis, biopsies were conducted to confirm the irAEs.

For all patients admitted to the ICU, type of malignancy, data on ICI treatment, irAE treatment, ICU admission, sequential organ failure assessment (SOFA) score, and survival outcomes were collected. The SOFA score is used to assess the severity of organ dysfunction in critically ill patients at the ICU, which is also used as a predictor for mortality based on six organ systems. SOFA scores range from 0 to 24 with higher scores indicating a worse prognosis.^18^ Overall survival (OS) was defined as the time from ICU discharge to death by any cause and follow‐up data was collected until July 2023. Data were extracted from the electronic medical records Hix (Chipsoft, Amsterdam, the Netherlands) and Metavision (iMDsoft, Dedham, Massachusetts, The United States). The conduct of this observational study was approved by the Investigational Review Board of the NKI‐AvL and the need for written informed consent was waived.

For quantitative data, results were expressed as medians with interquartile ranges. For categorical data, frequencies and percentages were used. OS was estimated using the Kaplan–Meier method and the median OS including the 95% confidence interval (CI) was reported. Statistics were performed in R version 4.2.1. (R Foundation for Statistical Computing, Vienna, Austria).

## 3 RESULTS

Over the study period, 5561 patients received at least one ICI administration at our institute, of which 32 patients (0.6%) were admitted to the ICU due to irAEs. The characteristics of this patient population are depicted in Table 1. Twenty patients (63%) were treated with anti‐PD‐1 plus anti‐CTLA‐4 treatment, whereas 12 patients (37%) were either treated with anti‐CTLA4 or anti‐PD‐1/PD‐L1 monotherapy. The median duration of ICU stay was 3 days. The median SOFA score on the first day of ICU stay was three.

**TABLE 1:** Characteristics of patients admitted to the intensive care unit (ICU) due to immune‐related adverse events (irAE) of immune checkpoint inhibitors (ICIs).

	Patients N = 32 (%)
Age, years	
Median (IQR)	66 (58–72)
Male sex	26 (81)
ECOG PS at time of start treatment	
0	17 (53)
1	12 (38)
NA	3 (9)
ICI used	
Atezolizumab	1 (3)
Durvalumab	1 (3)
Ipilimumab	3 (9)
Nivolumab	4 (13)
Nivolumab + ipilimumab	20 (63)
Pembrolizumab	3 (9)
Type of cancer	
Melanoma	17 (53)
Lung	5 (16)
Urothelial	3 (9)
Renal	2 (6)
Colorectal	2 (6)
Mesothelioma	2 (6)
Breast	1 (3)
Type of irAE	
Gastrointestinal	8 (25)
Ketoacidosis	8 (25)
Myocarditis and myositis	4 (13)
Myocarditis	4 (13)
Myositis	2 (6)
Nephritis	3 (9)
Pneumonitis	2 (6)
Myelitis	1 (3)
Time from ICI initiation until hospital admission due to irAE (days)	
Median (IQR)	44 (29–205)
Duration of hospital admission (days)	
Median (IQR)	15 (7–24)
Duration of ICU admission (days)	
Median (IQR)	3 (2–6)
SOFA score, day 1	
Median (IQR)	3 (1–5)
SOFA score, highest during ICU stay	
Median (IQR)	4 (2–6)

Abbreviations: ECOG PS, Eastern Cooperative Oncology Group performance status; IQR, interquartile range; SOFA, sequential organ failure assessment.

The observed irAEs were de novo diabetes‐related ketoacidosis (*n* = 8), immune‐related (IR) gastrointestinal toxicity (*n* = 8), myocarditis or myositis (*n* = 10), nephritis (*n* = 3), pneumonitis (*n* = 2), and myelitis (*n* = 1). The duration from the first ICI administration until hospitalization due to irAEs varied between patients. Noteworthy, most patients with myocarditis, myositis, and nephritis developed irAEs after one or two ICI administrations. In addition to the irAE contributing most to ICU admission, some patients experienced multiple types of irAEs. For example, three patients with diabetes‐related ketoacidosis also experienced IR‐gastrointestinal toxicity or hepatitis. In addition, a distinction between myocarditis and myositis as the main irAE contributing to ICU admission was not always clear.

**FIGURE 1:**
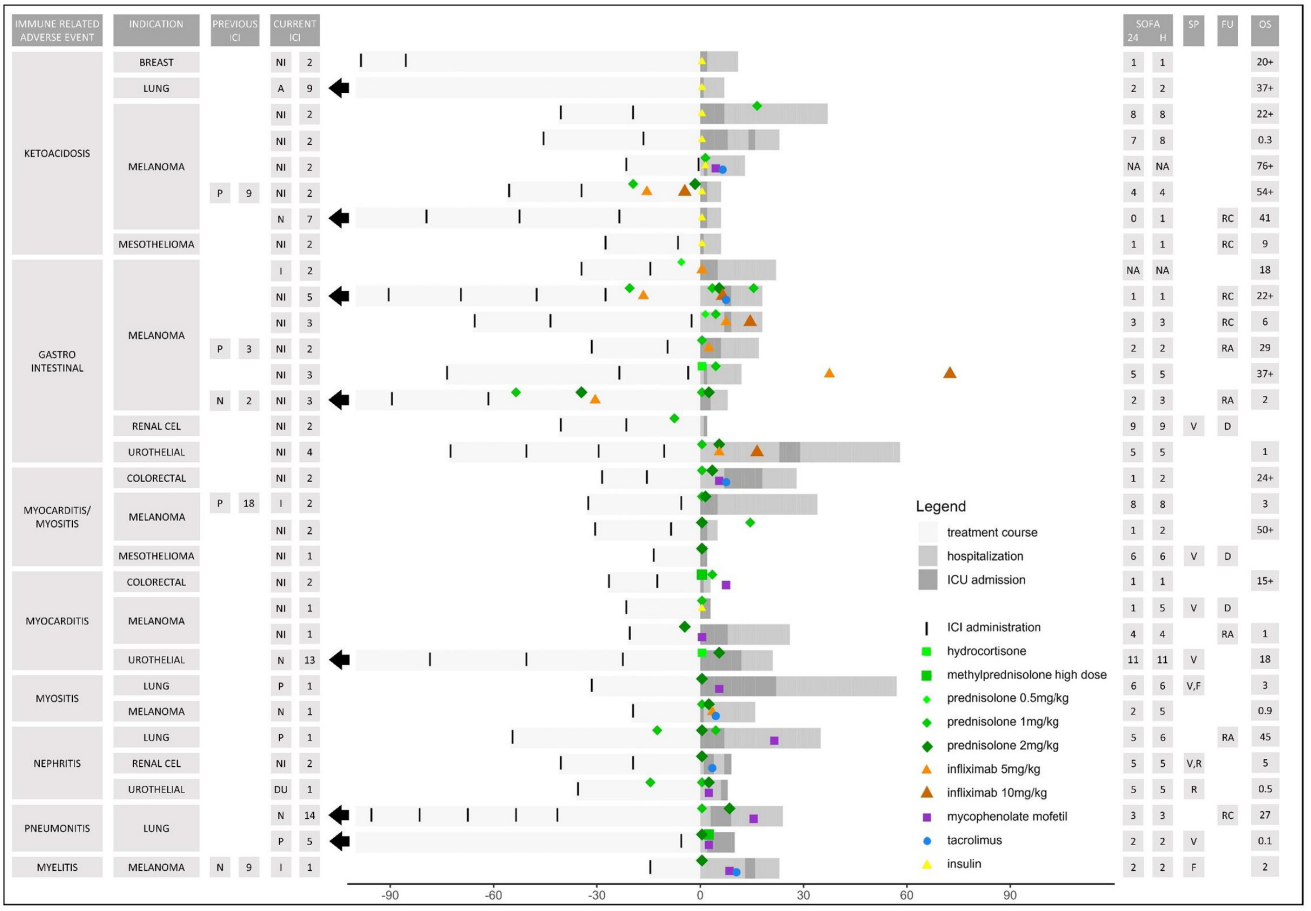
Overview of treatment course of patients administered to the intensive care unit (ICU) due to immune‐related adverse events of immune checkpoint inhibitors (ICIs) in days. One row represents one patient: the x‐axis shows time in days in which the black arrow indicates that the current ICI treatment was initiated longer ago. Previous and current ICI: type of ICI administered including the number of administrations. Atezolizumab (A), durvalumab (DU), ipilimumab (I), nivolumab (N), nivolumab + ipilimumab (NI), pembrolizumab (P). SOFA: SOFA score in the first 24 h of ICU stay (SOFA 24) and highest score during (first) ICU stay (SOFA H). SP: support in the form of mechanical ventilation (V), renal replacement therapy (R), and plasmapheresis (F). FU: follow‐up information including death during ICU stay (D), readmission (RA), and rechallenge with ICI (RC). OS: overall survival of patients discharged from the ICU in months with ‘+’ indicating that the patient was censored.

The treatment of these irAEs for each patient is depicted in Figure 1. The eight patients with de novo diabetes‐related ketoacidosis received treatment with insulin, of which three also received immunosuppressive therapy. In total, twenty seven patients (84%) received treatment with corticosteroids. In addition to corticosteroid treatment, eight patients (25%) received treatment with infliximab, which was mostly given in patients with IR‐gastrointestinal toxicity. Treatment with tacrolimus was administered in six patients (19%) and treatment with mycophenolate mofetil was administered in six patients (19%). In eight cases, patients were already started on corticosteroid treatment and other immunosuppressive treatment before hospital admission. In addition, seven patients (22%) required mechanical ventilation at the ICU, whereas two patients (6%) received renal replacement therapy and two patients (6%) received plasmapheresis.

**FIGURE 2:**
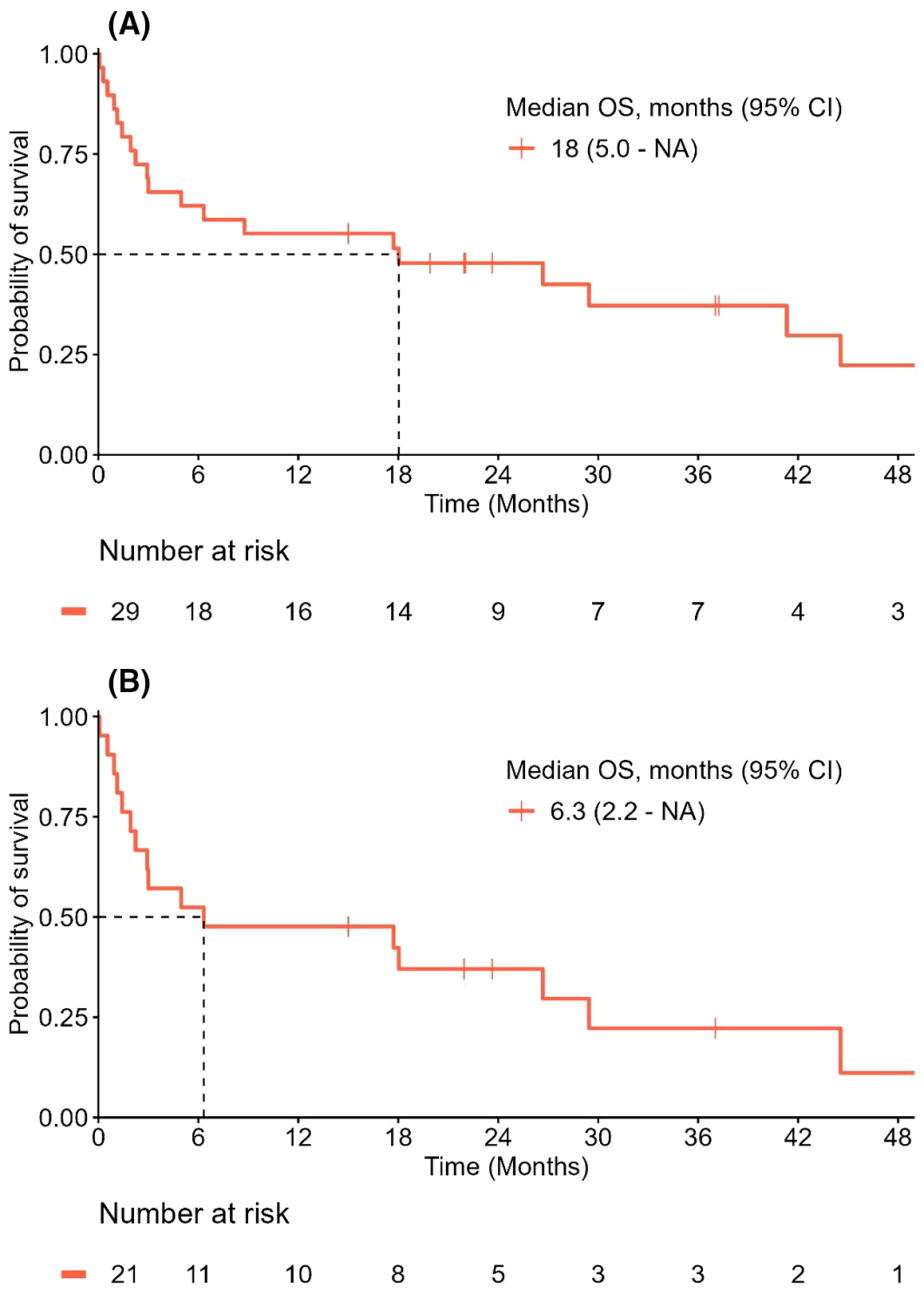
Overall survival (OS) of (A) patients from the time of intensive care unit (ICU) discharge till death and (B) patients without de novo diabetes‐related ketoacidosis. CI, confidence interval; NA, not available.

Three patients (9%) died during ICU admission. One patient died due to haemorrhagic shock caused by large duodenal ulcers. The second patient died due to a myocarditis‐related irreversible cardiogenic shock. The third patient decided to cease all treatment as a long recovery would be needed due to a combination of myositis and myocarditis, whereas no options remained to treat the underlying disease (mesothelioma). These three patients received only one or two cycles of anti‐PD‐1 plus anti‐CTLA‐4 treatment. Median OS of patients discharged from the ICU was 18 months (95% CI, 5.0—NA) (Figure 2A). Median OS of this patient population excluding patients with de novo diabetes‐related ketoacidosis was 6.3 months (95% CI, 2.2—NA) (Figure 2B).

No patients died on the general wards after ICU discharge, whereas six patients (19%) died within a month after hospital discharge. These patients had a poor prognosis, which was often due to a combination of the irAE and disease progression. An additional six patients (19%) died within 6 months after hospital discharge.

Four patients (13%) were readmitted within 30 days of discharge. In three of these patients, the readmission was related to the irAEs, whereas during hospital readmission additional immunosuppressiive treatment was administered. The other readmitted patient developed an infection, which was possibly related to the immunosuppressive treatment.

There were five patients (16%) who received a different ICI prior to the ICI treatment on which they developed irAEs. In all cases, no signs of irAEs were observed during the first ICI treatment line. Additionally, five patients (16%) were rechallenged with ICI treatment after ICU admission. Three patients were rechallenged with anti‐PD‐1 treatment and no recurrent irAEs were observed, whereas two patients were rechallenged with anti‐CTLA‐4 monotherapy or combination therapy, who did experience recurrent irAEs. These two patients initially presented with de novo diabetes‐related ketoacidosis, whereas during the rechallenge they presented with immune‐related hypophysitis and hepatitis. Both patients were treated with prednisolone.

## 4 DISCUSSION

This retrospective study determined the characteristics, treatment patterns and survival outcomes of patients admitted to the ICU due to irAEs from ICIs. The incidence of irAEs leading to ICU admission in patients treated with ICIs was low (0.6%), whereas ICU mortality was limited.

The frequency of irAEs leading to ICU admission in our study was much lower than the incidence of grade 3 or higher irAEs reported in the literature.^12^, ^19^ Especially the frequency of IR‐gastrointestinal toxicity leading to ICU admission was low compared to the incidence of grade 3 or higher colitis that occurs in 1–9% of patients depending on whether ICI monotherapy or combination therapy was given.^19^ Good awareness of irAEs, instructing patients to call at the first signs of a suspected IR‐gastrointestinal toxicity and immediate treatment initiation with corticosteroids may, therefore, prevent the worsening of irAEs and therefore also ICU admissions.

Consistent with the literature, anti‐CTLA‐4 treatment with or without anti‐PD‐1 treatment is associated with a higher risk of the development of irAEs leading to ICU admission compared to anti‐PD‐1/PD‐L1 monotherapy in our study.^12^ This higher risk to develop irAEs was also observed in our study within individuals who were treated with multiple ICI treatments. Patients who received anti‐PD‐1/PD‐L1 treatment prior to anti‐CTLA‐4 treatment with or without anti‐PD‐1 treatment, did not develop irAEs on the prior ICI treatment. In addition, patients who were rechallenged with anti‐PD‐1/PD‐L1 monotherapy did not develop irAEs, whereas patients rechallenged with anti‐CTLA‐4 treatment with or without anti‐PD‐1 treatment, did develop irAEs.

Regarding the onset of different types of irAEs, immune‐related myocarditis, myositis, and nephritis had a fast onset after ICI initiation. This fast onset was also observed for myositis in a study by Touat et al., in which the median onset was 25 days.^20^ For nephritis; however, different studies reported means or medians varying from 3 till 9 months after ICI initiation.^21^

In two other studies investigating ICU admission due to irAEs from ICI treatment, pulmonary irAEs were most common, which were respectively 28% and 64% of irAEs leading to ICU admission.^13^, ^16^ This differed from our results in which de novo diabetes‐related ketoacidosis and IR‐gastrointestinal toxicity were observed most frequently. A potential explanation for the high incidence of de novo diabetes‐related ketoacidosis may be due to a different treatment policy for de novo diabetes‐related ketoacidosis between the hospitals, as other hospitals may treat ketoacidosis at the general ward instead of the ICU. The ICU mortality in our study was lower compared to these two studies, which were 17% and 22%, respectively, whereas the SOFA score on the first day of ICU admittance was similar between our study and the study of Joseph et al. In addition, OS of patients admitted to the ICU due to irAEs was similar compared to the study of Joseph et al., which seemed to be longer compared to ICU admittance due to other reasons such as disease progression.^13^

There are several limitations in our study, which are inherent to the retrospective nature of this study and the low number of patients admitted to the ICU due to irAEs. Due to the low number of patients, it was not possible to compare the survival of patients with different types of irAEs. However, one can imagine that patients with de novo diabetes‐related ketoacidosis will have a better prognosis compared to other irAEs that need to be managed with immunosuppressive treatment. Therefore, additional studies are needed to study the survival outcomes between different types of irAEs. In addition, the fraction of patients needing ICU admission due to irAEs may be slightly underestimated, due to the possibility of ICU admittance at another hospital or a loss to follow‐up.

In conclusion, the incidence of irAEs leading to ICU admission in patients treated with ICIs was low in this study. ICU mortality due to irAEs was low and a subset of this patient population even had long‐term survival. Therefore, awareness of irAEs among all physicians involved in immunotherapy treatment, immediate initiation of corticosteroid treatment upon suspicion of irAEs and continuous multidisciplinary communication between the critical care and the oncology team is essential for the optimal treatment of this patient population.

## AUTHOR CONTRIBUTIONS

**Lishi Lin:** Conceptualization (equal); data curation (lead); formal analysis (lead); methodology (equal); writing – original draft (lead). **Aletta P. I. Houwink:** Conceptualization (equal); data curation (supporting); formal analysis (supporting); methodology (equal); writing – review and editing (equal). **Jolanda M. van Dieren:** Data curation (supporting); formal analysis (supporting); writing – review and editing (equal). **Esther K. Wolthuis:** Writing – review and editing (equal). **Johannes V. van Thienen:** Writing – review and editing (equal). **Michiel S. van der Heijden:** Writing – review and editing (equal). **John B. A. G. Haanen:** Writing – review and editing (equal). **Jos H. Beijnen:** Supervision (supporting); writing – review and editing (equal). **Alwin D. R. Huitema:** Conceptualization (equal); methodology (equal); supervision (lead); writing – review and editing (equal).

## CONFLICT OF INTEREST STATEMENT

L. Lin, A.P.I. Houwink, J.M. van Dieren, E.K. Wolthuis, J.V. van Thienen and A.D.R. Huitema declare no conflict of interest. M.S. van der Heijden has received research grants (paid to the institute) from BMS, AstraZeneca, Roche and 4SC and has served as an advisor (fees paid to the institute) to Roche, Pfizer, Astellas, Astra Zeneca, Merck Sharp and Dome, BMS, and Janssen. J.B.A.G. Haanen reports an advisory role for AZ, Achilles Therapeutics, BioNTech, Bristol‐Myers Squibb, CureVac, Imcyse, Immunocore, Instil Bio, Iovance Biotherapeutics, Ipsen, Molecular Partners, MSD Oncology, Neogene Therapeutics, Novartis, PokeAcell, Roche/Genentech, Sanofi, Scenic, Third Rock Ventures and T‐Knife; has received research funding (paid to the institute) from Amgen, Asher Biotherapeutics, BioNTech, Bristol‐Myers Squibb, MSD, Neon Therapeutics, Novartis, Sastra Cell Therapy; and is stock owner of Neogene Therapeutics. J.H. Beijnen is a part‐time employee and (indirect) shareholder of Modra Pharmaceuticals BV. He is (partly) patent holder of oral taxane formulations which are clinically developed by Modra Pharmaceuticals BV (a spin‐off company of the Netherlands Cancer Institute), not related to this work.

## ETHICS STATEMENT

Institutional Review Board of the Netherlands Cancer Institute—Antoni van Leeuwenhoek.

## Data Availability

The data generated and analyzed during the current study are available from the corresponding author on reasonable request.
